# Six-reflection meV-monochromator for synchrotron radiation

**DOI:** 10.1107/S0909049511017535

**Published:** 2011-05-26

**Authors:** T. S. Toellner, A. Alatas, A. H. Said

**Affiliations:** a Advanced Photon Source, Argonne National Laboratory, Argonne, IL 60439, USA

**Keywords:** high resolution, monochromator, X-ray

## Abstract

An in-line monochromatization scheme suitable for 10–40 keV synchrotron radiation is presented based on the use of six crystal reflections that achieves meV and sub-meV bandwidths with high efficiency. The theoretical spectral efficiency surpasses all previous multicrystal designs and approaches that of single room-temperature back-reflecting crystals. This article presents the designs of two such devices along with their theoretical and measured performances.

## Abstract 2

A design is presented for a cryogenically stabilized monochromator for 10–40 keV synchrotron radiation that uses six crystal reflections to achieve a meV-bandpass with high efficiency.

## 1. Introduction   

High-resolution X-ray spectroscopies that use synchrotron radiation in the 10–40 keV energy range require a monochromatic beam with a milli-electronvolt bandwidth. Spectroscopies such as inelastic X-ray scattering (Burkel, 1991 ▶) and nuclear resonant scattering (Gerdau & de Waard, 1999 ▶) involve measurements with low signal rates and long data-collection times. Consequently, improving the available spectral flux (photons s^−1^ meV^−1^) directly impacts the utility of these techniques. Improving the spectral flux can be done by either increasing the synchrotron radiation source strength or by increasing the efficiency of the monochromatization scheme.

Monochromatization of synchrotron radiation to a milli-electronvolt bandwidth usually involves the use of crystal reflections with large Bragg angles (>80°). Along with the narrow energy filtering that such reflections offer is a narrow angular acceptance (<1 µrad) that makes them inefficient when used with the substantially more divergent (∼10 µrad) synchrotron radiation. One exception is near-back Bragg reflections (Θ_B_ ≃ 90°), which have a large angular acceptance but restrict the design energy to match the back-reflection energy (Verbeni *et al.*, 1996 ▶; Baron *et al.*, 2001 ▶). Near-back Bragg reflections have high efficiency, but also redirect the beam back alongside the incident beam and require active temperature control to perform energy scans. An alternative to near-back Bragg reflections is to use a series of Bragg reflections (Θ_B_ < 90°) to perform the monochromatization (Toellner, 2000 ▶). This approach usually has difficulty matching the theoretical efficiency of a single near-back Bragg reflecting crystal, but offers a number of advantages. It offers greater freedom to choose the design energy, which is the paramount criteria for nuclear resonant spectroscopy, because one needs to monochromatize the synchrotron radiation at a nuclear resonance energy. It also offers greater suppression of spectral components outside the nominal bandwidth, the possibility to direct the beam into the forward direction, and the ability to change or scan the energy rapidly through active mechanical control (crystal rotation). Furthermore, a multicrystal in-line design allows one to position subsequent focusing optics much closer to the source where beam sizes are smaller, thus making it possible to achieve full-beam focusing with reduced aberrations and greater efficiency. Consequently, for high-resolution X-ray spectroscopy requiring few-micrometer focal-spot sizes, the multicrystal design offers the possibility of greater overall spectral intensity at the sample position.

Here we present a new design for a high-resolution monochromator (HRM) that employs six silicon crystal reflections to achieve high efficiency. When combined with cryogenic stabilization (Toellner *et al.*, 2006 ▶), this design offers a theoretical efficiency that is comparable with that of a single near-back Bragg reflection operating at room temperature. We constructed two cryogenically stabilized six-reflection meV-monochromators. Both HRMs have a tunability range of approximately 200 eV.

One monochromator, to be referred to as ‘HRM-1’, is designed to operate at 21.657 keV corresponding to the back-reflection energy for a silicon (18 6 0) pixelated spherical analyzer. This monochromator is part of a meV-resolution inelastic X-ray scattering spectrometer that is installed at beamline 3-ID of the Advanced Photon Source (Sinn *et al.*, 2001 ▶) and has been operational since 2004. Another monochromator, to be referred to as ‘HRM-2’, is designed to reach two energies: 23.725 keV, corresponding to the back-reflection energy for a silicon (12 12 12) pixelated spherical analyzer, and 23.880 keV, corresponding to a nuclear resonance energy in ^119^Sn. This monochromator is part of a meV-resolution inelastic X-ray scattering spectrometer that is installed at beamline 30-ID of the Advanced Photon Source and has been operational since 2007. It is designed to operate also at a nuclear transition energy (23.880 keV) to allow, in addition, nuclear resonant scattering measurements from materials containing ^119^Sn.

## 2. Design   

The principal problem with designing an efficient HRM without using a near-back Bragg reflection resides in the mismatch between the angular divergence of currently available synchrotron radiation sources and the intrinsic angular acceptance of small-energy-width crystal reflections. For multicrystal monochromators, one can take advantage of asymmetrically cut (non-zero angle between surface normal and lattice vector) crystals to tailor the angular acceptance of crystal reflections and the divergence of diffracted X-rays (Renninger, 1961 ▶; Kohra, 1962 ▶) to increase efficiency and improve energy resolution of meV-bandwidth monochromators (Ishikawa *et al.*, 1992 ▶; Toellner *et al.*, 1992 ▶). Selection of the crystal lattice reflections (**H**_*i*_) and the asymmetry angles (α_*i*_) to use as part of a HRM that achieves maximal efficiency is guided by the desired energy bandwidth (in our case, ∼1 meV) and practical limitations such as crystal size. For the present designs, diffraction footprints are limited to be less than 6 cm. Optimal X-ray transmission is achieved by requiring that monochromatic rays diffracting from any crystal are diffracted from all subsequent crystals. This requirement leads to constraints on angular acceptances of crystal reflections and thus guides the choice of lattice reflections and asymmetry angles. These constraints can be expressed as a set of matching conditions 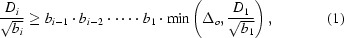
where *D*_*i*_ is the intrinsic Darwin width and 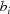 = 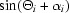/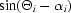 is the asymmetry parameter for the *i*th reflection. Δ_*o*_ is the incident beam divergence for monochromatic rays. Satisfying these matching conditions while obtaining the desired energy bandwidth forms the basis for the monochromator design.

**Figure 1:**
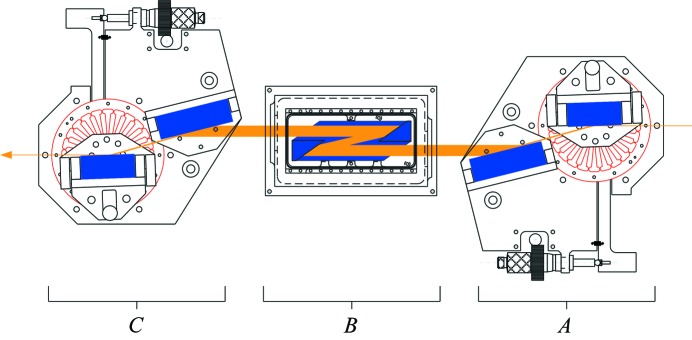
Design of a six-reflection cryogenically stabilized high-resolution monochromator. *A*, *B* and *C* refer to crystal-reflection pairs described in the text. Crystal-reflection pair *B* is cooled to 123 K, while *A* and *C* are maintained at room temperature.

We constructed two HRMs based upon a six-reflection design of the type (+*A*, −*A*, −*B*, +*B*, +*C*, −*C*) shown in Fig. 1 ▶. We employ pairs of reflections to insure that the transmitted beam is directed into the forward direction independent of energy alignment. The bulk of the monochromatization is performed by the second pair of reflections denoted by ‘*B*’, which are silicon crystal reflections with a large Bragg angle (∼83°) and an intrinsically narrow energy acceptance. These two reflections are formed from opposing parallel surfaces within a monolithic block of crystalline silicon. Thus, they have reciprocal asymmetry parameters (*b*_3_ = *b*_4_^−1^). The diffracting surfaces are cut asymmetrically to reduce their energy acceptance to approximately 1 meV when the silicon is cooled to 123 K. The actual choice for the asymmetry parameter for this pair of reflections is obtained from simulations, but an approximate value can be obtained from 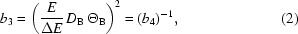
where *E* is the X-ray energy, Δ*E* is the desired energy bandwidth, and *D*_B_ and Θ_B_ are the Darwin width and Bragg angle of the reflection denoted by ‘*B*’, respectively. This expression assumes the beam divergence incident on the first ‘*B*’ crystal is reduced to a value small compared with 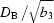. Note that by choosing the appropriate asymmetry parameter one can achieve energy bandwidths that are smaller or larger than the intrinsic energy bandwidth of the crystal reflection.

Operating this crystal pair at 123 K, which corresponds to the zero-thermal-expansion temperature in silicon (Touloukian *et al.*, 1977 ▶), offers significant advantages in terms of efficiency and stability (Toellner, 2000 ▶). Only the second pair of crystal reflections is cooled in a specially designed low-vibration cryostat (Toellner *et al.*, 2006 ▶). Unlike earlier cryostat versions, the current design incorporates a mechanical pump to flow the helium cooling gas through the cryostat and through a heat exchanger immersed in a liquid-nitrogen bath in a closed cycle. Pump-induced pulsations within the gas stream that may cause vibrations within the cryostat are suppressed by the use of pulsation dampeners.

The small energy acceptance of this pair is accompanied by a narrow angular acceptance and this necessitates the need for preceding collimation that reduces the angular divergence of the incident synchrotron radiation. This is achieved by the first pair of reflections denoted by ‘*A*’, which are low-Bragg-angle (Θ_B_ < 10°) silicon crystal reflections cut asymmetrically so they have a collimating effect on the diffracted synchrotron radiation. Both reflecting crystals are mounted on a weak-link mechanical assembly (Shu *et al.*, 2001 ▶) to allow them to share the same rotation axis. Unlike the previously published weak-link assembly, the current version uses a stepper-motor-driven micrometer head in series with a piezo-actuator to allow coarse and fine adjustments, respectively. By using two reflections to carry out the divergence reduction, it is possible to use very high reflectivity reflections with modest asymmetry angles, thus producing a very efficient beam collimator (Kikuta & Kohra, 1970 ▶; Matsushita *et al.*, 1971 ▶). For both monochromators the first pair of reflections combine to collimate the synchrotron radiation by a factor of ∼45. This reduces the beam divergence of the synchrotron radiation to approximately 0.2 µrad, which is less than the angular acceptance of the *B* crystal reflection pair (0.3–0.5 µrad) and significantly improves the overall efficiency.

A consequence of the divergence reduction created by crystal pair *A* is a beam expansion so that the component of the beam size in the diffraction plane is a factor of ∼45 larger than the incident beam. Owing to its large Bragg angle the second crystal pair needs to be made large enough to transmit this expanded beam size. The collimation is currently constrained by present-day vertical beam sizes available at synchrotron sources and the need to keep the monolithic crystal that forms the second pair of reflections to an acceptable size.

In our configuration, crystal pair *B* is in a (−, −) setting with respect to crystal pair *A*, but this actually has little impact on performance (resolution or efficiency) for the HRMs presented here owing to the fact that the crystal reflections chosen for the two pairs are highly dispersive already. In the general case though, the setting (+*A*, −*A*, −*B*, +*B*, +*C*, −*C*), as opposed to (+*A*, −*A*, +*B*, −*B*, +*C*, −*C*), will offer both greater resolution in cases where crystal pair *A* is chosen to be a higher-order reflection as has been demonstrated previously (Yabashi *et al.*, 2001 ▶), and smaller offsets between input and output beams.

After reflections *A* and *B* the transmitted beam has a ∼1 meV bandwidth, but is still expanded owing to the first collimation stage. The third pair of reflections, denoted ‘*C*’, compresses the beam back to its original size. The lattice reflections and asymmetry angles for both monochromators are given in Table 1 ▶. The net effect of all six reflections is to restore the beam direction, divergence and size. This results from both the use of crystal reflection pairs and the choice of asymmetry angles such that the product of all the asymmetry parameters is unity. The transmitted beam for both HRMs is vertically offset from the incident beam by approximately 3 cm, which is substantially less than what was obtained (11 cm) with a cryogenically stabilized ‘nested’ design (Toellner *et al.*, 2006 ▶). The operational parameters (Bragg angles and asymmetry parameters) for both monochromators are given in Table 2 ▶.

**Figure 2:**
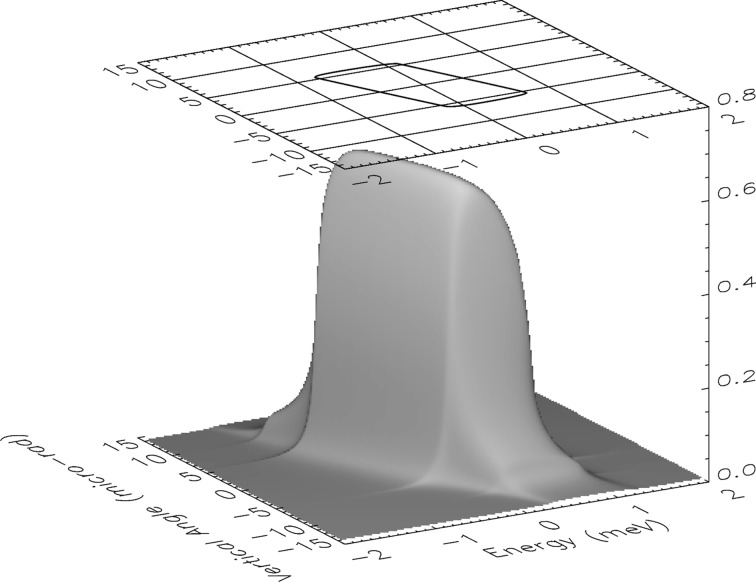
Calculated bare-optic transmission function of HRM-2 for 23.725 keV σ-polarized photons. The peak transmission is 64%. The contour at the top of the figure is drawn at 50% of this value, and represents a Dumond diagram in the input beam coordinates. The transmission functions at 23.880 keV as well as that for the other monochromator (HRM-1) are similar.

A simulation of the bare-optic transmission for σ-polarized radiation through HRM-2 (23.725 keV) assuming two-beam dynamical diffraction is presented in Fig. 2 ▶ as a function of energy and vertical angle of the incoming radiation. The contour plot displayed in Fig. 2 ▶ represents a Dumond diagram of the HRM in the input beam coordinates. Note that the peak transmission after all six reflections for the bare optic (*i.e.* air and beryllium windows not included) is 64%. The transmission functions at 23.880 keV as well as for HRM-1 are very similar.

Angular control of the crystals is an important aspect of the design. Each crystal assembly composed of a pair of reflections is mounted on a Kohzu KTG-15 goniometer and operated inside an enclosure to reduce external thermal influences. Energy scans over small energy ranges (∼200 meV) require rotating only the second pair of crystals by an amount given by 0.4040 µrad meV^−1^ (HRM-1 at 21.657 keV). For HRM-2, this energy-to-angle conversion is 0.4050 µrad meV^−1^ at 23.725 keV and 0.2744 µrad meV^−1^ at 23.880 keV. The angular acceptance of the third pair of reflections is 0.55 µrad for HRM-1 and approximately 0.44 µrad for HRM-2, while the beam divergence after the second pair of reflections is approximately 0.2 µrad for both HRMs. This results in a very sensitive angular alignment requiring 0.05 µrad stability that is easily corrupted resulting in a significant drop in transmission. To maintain this alignment, a low-profile piezo-driven rotation stage with zero backlash was designed and inserted between the third crystal-pair assembly and its goniometer, and a control loop actively maintains the proper alignment using a downstream ion chamber as a reference signal. HRM-1 uses a simple software control algorithm operating at 1 Hz to maintain the alignment. Using a slightly different approach, HRM-2 employs a lock-in amplifier and requires oscillating the crystal assembly at 10 Hz to maintain the alignment (Stoupin *et al.*, 2010 ▶). Both approaches work reasonably well and automatically maintain alignment and mitigate any variation in transmission.

## 3. Testing   

We tested HRM-1 at the 3-ID undulator beamline of the Advanced Photon Source. Synchrotron radiation from this beamline’s two undulators (2.7 cm magnetic period), totaling a length of 4.8 m, passes through a water-cooled beryllium compound refractive lens (CRL) before monochromatization at 21.657 keV using a water-cooled diamond (1 1 1) double-crystal premonochromator. The CRL produces a partial vertical collimation of the raw synchrotron radiation to reduce the angular divergence of the X-rays to be within the narrow angular acceptance of the diamond (1 1 1) premonochromator (Zhao *et al.*, 2002 ▶). After the premonochromator the energy bandwidth of the photon beam is 1.4 eV full width at half-maximum (FWHM). The HRM is placed after the premonochromator in such a way that the first crystal reflection is in a (+, +) scattering geometry with respect to the second crystal of the premonochromator. This has two advantages. First, it gives a more flat spectral response after the first pair. This is a useful feature, as it allows single-axis energy-scanning of the second crystal pair over an energy range of a few hundred milli-electronvolts with little change in the transmitted flux. Second, it results in a narrower energy bandwidth after the first crystal pair, and thus a reduced heat load on the second pair, which reduces the cooling power needed by any low-vibration cryostat.

The two most important performance metrics for any HRM are transmitted energy bandwidth and spectral efficiency. The energy bandwidth transmitted by HRM-1 is determined from a measurement of the instrumental resolution function of an inelastic X-ray scattering spectrometer, of which the monochromator is a part. The other part of the spectrometer is a silicon (18 6 0) pixelated spherical analyzer that operates in near back-reflection geometry (Sinn, 2001 ▶). The analyzer has a 6 m radius of curvature and is operated in a Rowland condition with respect to the sample position and a CdTe X-ray detector. The analyzer has a theoretical energy acceptance of 1.8 meV in this geometry. By using a sample with a known dynamical structure factor, and aligning the analyzer to the maximum in the structure factor that gives strong elastic scattering, we obtain counting rates sufficient for a determination of the resolution function of the overall spectrometer. The energy bandwidth of the spectrometer’s resolution function is 2.2 meV FWHM. After deconvolution, we obtain an energy bandwidth (Δ*E*_meas_) of 1.25 ± 0.1 meV FWHM for the contribution from HRM-1. This agrees fairly well with the theoretical result (Δ*E*_theory_) of 1.15 meV obtained from simulations of HRM-1 when combined with this undulator source and upstream optics.

Measuring the spectral efficiency involves determining the ratio of fluxes and the ratio of energy bandwidths before and after the HRM. The fluxes were measured using a calibrated silicon photodiode. The energy bandwidth of the premonochromated beam is measured by scanning the energy of HRM-1 to determine the spectral distribution of the incident beam. An incident premonochromated X-ray beam of approximately 1.4 × 10^13^ photons s^−1^ (*F*_0_) in a 1.4 eV FWHM bandwidth (Δ*E*_inc_), or a spectral flux of 1.0 × 10^13^ photons s^−1^ eV^−1^, is reduced to an X-ray beam of 5.4 × 10^9^ photons s^−1^ (*F*) in a 1.25 meV FWHM bandwidth, or a spectral flux of 4.3 × 10^12^ photons s^−1^ eV^−1^. We define the spectral efficiency as the ratio of the spectral flux after the HRM to the available spectral flux before the HRM using the full premonochromated beam. Using this definition, we obtain a measured spectral efficiency (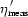) of 43%. This compares favorably with the theoretical result (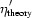) of 51%. Note that these results, both measured and theoretical, include losses due to absorption in air and beryllium windows, which have a combined transmission of 90%. To allow meaningful comparisons between different optical designs, it is useful to remove these losses and others that may be mitigated in an improved implementation. We refer to the result as the ‘bare-optic efficiency’ for the design. By removing these losses, one obtains a measured bare-optic efficiency (η_meas_) of 48% and a theoretical bare-optic efficiency (η_theory_) of 57%.

**Figure 3:**
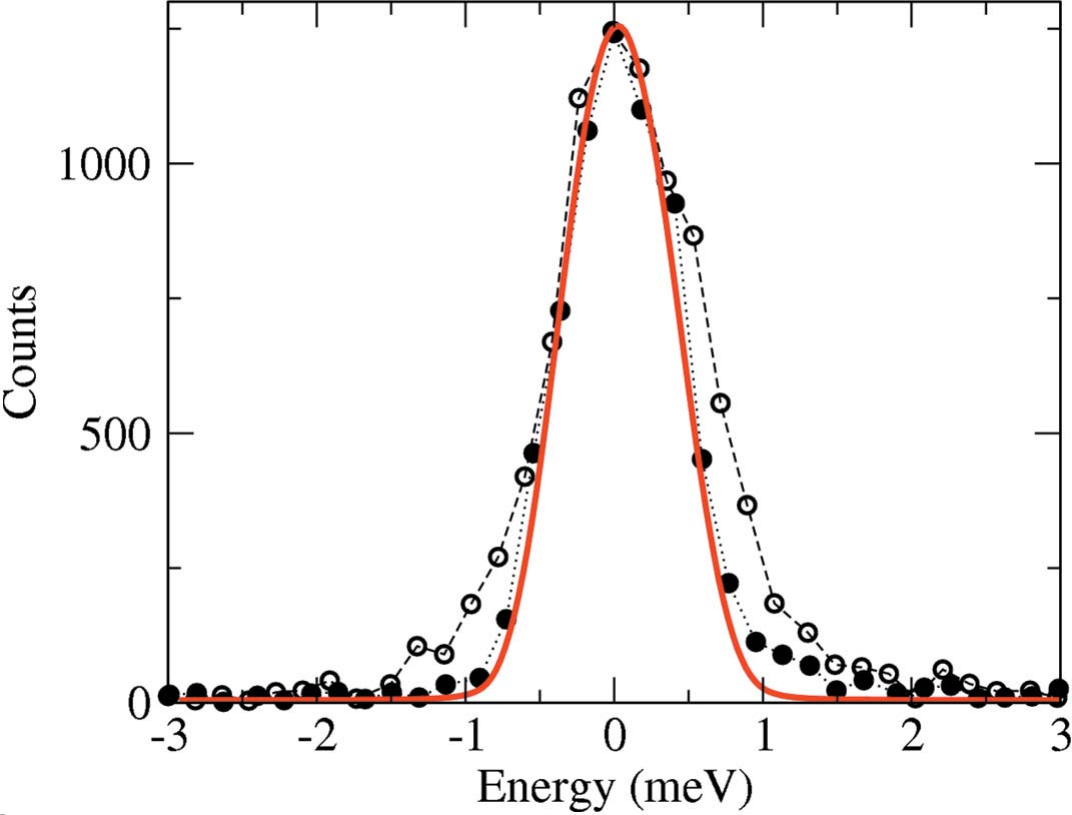
Resolution function of one of the monochromators (HRM-2) measured at 23.880 keV using nuclear resonant scattering from ^119^SnO_2_. The resolution function is measured under normal operating conditions with a cooling-gas flow rate of 0.13 l s^−1^ (open circles), and with a reduced cooling-gas flow rate of 0.017 l s^−1^ (filled circles). The solid line is the expected result assuming two-beam dynamical diffraction and no vibrations.

We tested HRM-2 at the 30-ID undulator beamline of the Advanced Photon Source. The 30-ID beamline is identical in terms of upstream beamline components to the 3-ID beamline described above. The performance of HRM-2 is characterized in a very similar way, except that, because it is able to operate at 23.880 keV, corresponding to a nuclear resonant transition in ^119^Sn that has a resonance linewidth of 25 neV, we measure the energy resolution function directly using elastic nuclear resonant scattering from a ^119^SnO_2_ powder. Owing to the greater precision that is possible with this method, we measure the energy resolution function with the cryostat operating under normal conditions with a helium-gas flow rate of 0.13 l s^−1^ and obtain a transmitted bandwidth of 1.1 meV FWHM. Reducing the helium-gas flow rate in the cryostat to 0.017 l s^−1^ produces a resolution function with a transmitted bandwidth of 0.9 meV FWHM, which is also the theoretical value. The cryostat has sufficient thermal inertia that the temperature does not change significantly during this test and no energy drift is observable. Thus, by reducing flow-induced vibrations, HRM-2 produces its theoretical energy bandwidth. Both measured resolution functions along with the simulation that assumes two-beam dynamical diffraction and no vibrations and has a FWHM of 0.9 meV are shown in Fig. 3 ▶.

The additional broadening for 23.880 keV X rays (0.2 meV) observed at the higher flow rate is an energy-alignment instability resulting from gas-flow-induced angular vibrations of crystal pair *B*. From the measured broadening the vibrations have an angular distribution (assumed Gaussian) of 0.18 µrad FWHM. The impact of the angular vibrations on the energy instability resulting in an effective energy broadening will be less (greater) if the operating energy is less (greater) owing to an increasing (decreasing) energy-to-angle conversion factor. Consequently, for HRM-2 operating at 23.725 keV, the angular vibrations contribute only an additional 0.1 meV to the FWHM of the otherwise theoretical energy bandwidth.

A measurement of spectral efficiency for HRM-2 is carried out in the same manner as for HRM-1 described above. The theoretical and measured performances for both HRMs are presented in Table 3 ▶. Owing to the energy-alignment instability that is not intrinsic to the monochromator’s crystal configuration and, in principle, could be mitigated in a future implementation, the tabulated bare-optic efficiency for HRM-2 is calculated assuming theoretical bandwidth along with no losses due to air or beryllium windows. Also note that flux values (*F*_0_ and *F*) given in Table 3 ▶ are current values and may change as a beamline and its source experience improvements and/or upgrades.

## 4. Discussion   

Measured energy bandwidths agree fairly well with the theoretical energy bandwidths for both HRMs. The minor discrepancy for HRM-2 has been shown to be related to an energy-alignment instability, but it is possible, and perhaps likely, that HRM-1 also suffers from the same instability because the designs are essentially identical. To obtain better agreement will require mitigating this energy-alignment instability that originates from gas-flow-induced vibrations. This instability is absent in a previous version of a low-vibration cryostat that did not operate in a closed cycle and thus had no pump producing pulsations in the gas flow. As the current design employs a pump to allow closed-cycle operation and pulsation dampeners to suppress any pressure waves in the gas stream, we suspect that our design of the dampeners is inadequate.

One of the reasons this six-reflection design is more efficient than previous in-line designs is that the presence of the third pair for beam compression allows greater freedom for optimizing the first two crystal pairs for resolution and efficiency without having to also maintain beam size. For example, the current design can be modified to produce even smaller energy bandwidths with little to no reduction in efficiency by increasing the asymmetry parameter on the fourth reflection. This has two consequences. First, it will necessitate a means to compensate the differing refractive shifts between the third and fourth crystal reflections while at cryogenic temperatures, perhaps with a weak-link mechanical structure. Second, it will alter the size of the output beam, because the product of all the asymmetry parameters will no longer be unity. If one starts from the current design, the incident beam size can be restored, if desired, while satisfying the matching conditions, by decreasing *b*_5_ while keeping the product *b*_4_ *b*_5_ constant. Other ways to reduce the energy bandwidth of the HRMs include increasing the order of the first pair of reflections (Yabashi *et al.*, 2001 ▶) and/or decreasing their asymmetry parameters.

**Figure 4:**
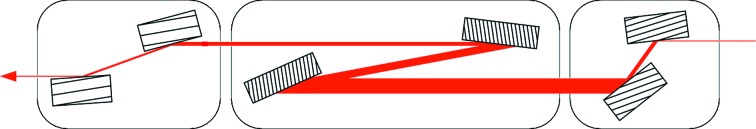
General crystal arrangement for a six-reflection monochromator that produces high spectral efficiency with improved energy resolution.

A more general design for the six-reflection monochromator that allows *b*_3_ ≠ *b*_4_^−1^ can achieve higher energy resolutions while maintaining high spectral efficiency and is shown in Fig. 4 ▶. Allowing this more general design, Table 4 ▶ presents design parameters for HRMs at various other X-ray energies corresponding to nuclear resonances: 14.413 keV (Fe^57^), 22.494 keV (Sm^149^) and 37.150 keV (Sb^121^). The design parameters presented in Table 4 ▶ are only representative. Optimal parameters depend upon the actual size and divergence of a particular X-ray beam. The quantity ΔΘ refers to the angular acceptance of the HRM in the diffraction plane, Δ*E* is the energy bandwidth (FWHM), and *T* is the peak bare-optic transmission. Actual energy bandwidths are somewhat source-dependent, so a range of energy bandwidths is given for each HRM.

In some cases it is beneficial to reduce the size and divergence of the X-ray beam incident on the HRM using an X-ray CRL upstream (Baron *et al.*, 1999 ▶). This can relax design criteria such as the size of crystal pair *B* and/or the amount of collimation required by crystal pair *A*, thus making a high spectral efficiency more readily attainable. This is especially important to consider for X-ray energies where the Bragg angle for crystal pair *B* is large (>85°) and/or the corresponding Darwin width is small (<0.2 µrad).

Theoretical bare-optic efficiencies (∼56%) for both HRMs are somewhat less than peak bare-optic transmissions (∼64%) owing to our operational definition of efficiency and to the fact that transmitted spectral components beyond the FWHM are suppressed because of multiple crystal reflections. This leads to resolution functions with reduced tails, which benefit spectroscopic measurements that attempt to resolve inelastic X-ray scattering excitations lying close to elastic lines. Furthermore, the peak bare-optic transmission for a six-reflection HRM is almost identical to the room-temperature theoretical reflectivity of just one of the crystal reflections that is part of crystal pair *B*. For example, the room-temperature reflectivity of the silicon (15 11 9) reflection that is part of crystal pair *B* in HRM-2 is 65%, while peak transmission for all six reflections of HRM-2 is 64%. Owing to the fact that crystal reflections in silicon with even-integer indices have higher reflectivities than odd-integer-indexed reflections with similar lattice spacings, constructing an HRM using even-integer-indexed silicon reflections for crystal pair *B* would be commensurately more efficient.

Measured spectral efficiencies currently are within 60–85% of their theoretical values. In the case of HRM-1, if the additional energy broadening is due to the energy instability mentioned above, the measured bare-optic efficiency would increase from its tabulated value of 48% to 52%, and this would then be within 90% of its theoretical value. In general, attaining the theoretical spectral efficiency requires using exceptionally high-quality single-crystal silicon and the utmost care in fabrication, mounting and alignment. The discrepancies between the measured and theoretical efficiencies result from a combination of the measured flow-induced energy broadening and perhaps crystal fabrication and/or mounting strain.

Apart from using even-integer-indexed crystal reflections and improving fabrication methods, efficiencies can be improved by operating an HRM in a vacuum or a low-pressure gas environment to mitigate X-ray losses owing to air absorption. Also, by operating in a low-pressure gas environment, one could employ windowless ionization detectors to monitor flux and avoid absorption in windows and air.

During inelastic X-ray scattering measurements it is sometimes beneficial or even necessary to have access to a much more intense beam for purposes such as sample alignment, downstream optics alignment or *in situ* crystal structure measurements of the sample under study. Owing to the nature of the six-reflection design, this can be achieved readily by switching between crystal pair *B* that produces a meV-bandwidth beam and a low-order crystal pair that produces a much larger bandwidth, but with substantially more flux. This will allow remote switching between a high-resolution X-ray beam and a low-resolution high-flux X-ray beam as needed and while maintaining beam position. For the current HRMs a pair of symmetric silicon (4 4 0) crystal reflections with the same vertical beam offset as the crystal pair *B* that it is replacing would produce approximately a 140 meV bandwidth beam with two orders of magnitude more flux. The more intense beam would allow faster alignments and faster ancillary measurements that facilitate sample investigations, but do not need high-energy resolution, *e.g.* crystal-structure-related measurements. This is especially useful for studies of samples under extreme conditions, such as at very high pressures and temperatures where samples are exceedingly small and/or produce very little scattering. This is a superb feature that adds versatility and can be used to improve the utility of the HRM towards more comprehensive measurements. 

## Figures and Tables

**Table 1:** Design parameters for the two high-resolution monochromators Reflections **H**_3,4_ are at 123K, while the others are at room temperature. Asymmetry angles (_*i*_) are in degrees.

	**H**_1,2_	**H**_3,4_	**H**_5,6_	_1_	_2_	_3_	_4_	_5_	_6_
HRM-1	(220)	(15113)	(220)	6.6	6.0	61.5	61.5	6.0	6.6
HRM-2	(311)	(15119)	(220)	6.8	6.8	52.9	52.9	5.8	5.8

**Table 2:** Operating parameters for the two high-resolution monochromators

	Energy	_1,2_	_3,4_	_5,6_	*b*_1_	*b*_2_	*b*_3_	*b*_4_	*b*_5_	*b*_6_
HRM-1	21657eV	8.573	83.367	8.573	0.13	0.18	1.5	0.65	5.6	7.6
HRM-2	23725eV	9.182	83.958	7.821	0.15	0.15	1.3	0.75	6.7	6.7
HRM-2	23880eV	9.122	81.107	7.770	0.15	0.15	1.5	0.66	6.8	6.8

**Table 3:** Expected and measured performances for both monochromators

	Energy (eV)	*F*_0_/*E*_inc_ (photons s^1^ eV^1^)	*F* (photons s^1^)	*E*_theory_ (meV)	*E*_meas_ (meV)	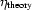 (%)	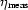 (%)	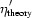 (%)	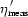 (%)
HRM-1	21657	1.4 10^13^/1.4	5.4 10^9^	1.15	1.25	57	48	51	43
HRM-2	23725	1.1 10^13^/1.6	2.4 10^9^	0.9	1.0	56	43	51	35
HRM-2	23880	1.1 10^13^/1.6	2.2 10^9^	0.9	1.1	56	40	51	29

**Table 4:** Design parameters and expected performances for six-reflection monochromators proposed at various energies Reflections **H**_3,4_ are at 123K, while the others are at room temperature. Asymmetry angles (_*i*_) are in degrees.

Energy (eV)	**H**_1,2_	**H**_3,4_	**H**_5,6_	_1_	_2_	_3_	_4_	_5_	_6_	*E* (meV)	(rad)	*T* (%)
14413	(422)	(1064)	(111)	17.3	17.3	69.9	69.9	0	3.7	1.01.4	15	64
22494	(400)	(1688)	(220)	9.2	8.2	70	70	2.9	6.1	0.81.0	14	73
37130	(422)	(3244)	(422)	6.9	5.9	0	0	6.4	6.4	0.200.22	6	59
